# NOTCH1 Mutations in Aortic Stenosis: Association with Osteoprotegerin/RANK/RANKL

**DOI:** 10.1155/2017/6917907

**Published:** 2017-01-26

**Authors:** Olga Irtyuga, Anna Malashicheva, Ekaterina Zhiduleva, Olga Freylikhman, Oxana Rotar, Magnus Bäck, Svetlana Tarnovskaya, Anna Kostareva, Olga Moiseeva

**Affiliations:** ^1^Federal Almazov Medical Research Centre, St. Petersburg, Russia; ^2^Institute of translational Medicine, ITMO University, St. Petersburg, Russia; ^3^St. Petersburg State University, St. Petersburg, Russia; ^4^Department of Medicine and Centre for Molecular Medicine, Karolinska Institutet, Stockholm, Sweden; ^5^St. Petersburg Polytechnic University, St. Petersburg, Russia; ^6^Department of Woman and Child Health and Centre for Molecular Medicine, Karolinska Institutet, Stockholm, Sweden

## Abstract

*Background*. The NOTCH pathway is known to be important in the pathogenesis of calcific aortic valve disease, possibly through regulators of osteoprotegerin (OPG), receptor activator of nuclear factor *κ*B (RANK), and its ligand (RANKL) system. The* purpose* of the present study was to search for possible associations between* NOTCH1* gene mutations and circulating levels of OPG and soluble RANKL (sRANKL) in patients with aortic stenosis (AS).* Methods*. The study was performed on 61 patients with AS including 31 with bicuspid and 30 with tricuspid aortic valves. We applied a strategy of targeted mutation screening for 10 out of 34 exons of the* NOTCH1* gene by direct sequencing. Serum OPG and sRANKL levels were assessed.* Results*. In total, 6 genetic variants of the* NOTCH1* gene including two new mutations were identified in the study group. In an age- and arterial hypertension-adjusted multivariable regression analysis, the serum OPG levels and the OPG/sRANKL ratio were correlated with* NOTCH1* missense variants. All studied missense variants in NOTCH1 gene were found in Ca(2+)-binding EGF motif of the NOTCH extracellular domain bound to Delta-like 4.* Conclusion*. Our results suggest that the OPG/RANKL/RANK system might be directly influenced by genetic variants of NOTCH1 in aortic valve calcification.

## 1. Introduction

Aortic stenosis (AS) due to tricuspid (TAV) and bicuspid (BAV) aortic valve calcification is the most frequent valvular heart disease and the third leading cause of adult heart disease [1]. There are currently no medical interventions capable of delaying or halting aortic stenosis progression. AS was previously considered as a degenerative aortic valve disease and hypothesized to be due to a passive accumulation of calcium binding to the aortic surface of the valve leaflet. However, several studies have now suggested that aortic valve calcification is an active biological process with a strong genetic component involving mechanisms similar to osteogenesis [2–6].

NOTCH is a key signaling pathway in development, ensuring crosstalk between different types of cells and their physiological differentiation [7], and is particularly important during cardiac valvulogenesis. In the vascular system, all NOTCH receptors (NOTCH1–NOTCH4) and ligands (Jag1 and Jag2 and Dll1, Dll3, and Dll4) are expressed, albeit at different levels and distinctly in different vascular cells and vessel types. The outcome of NOTCH activation is cell type and context dependent with multiple combinations of receptors and ligands that transduce different biological effects [8]. Several lines of evidence in addition suggest that the NOTCH pathway might be important in the adult heart, notably in the pathogenesis of calcific aortic valve disease [9, 10]. Furthermore, several studies on NOTCH-ligand binding have demonstrated the requirement for calcium-binding EGF domains of NOTCH as well as the presence of calcium for productive interaction [11–13].

However the exact mechanisms of NOTCH action in aortic valve calcification remain unknown and the existing evidence is rather controversial. Acharya et al. demonstrated through chemical inhibition that NOTCH1 has an inhibitory role on the development of CAVD [14]. Further, Nigam and Srivastava showed that NOTCH1 signaling specifically affects osteogenic pathways in VIC, preventing the progression of osteogenic calcification [15]. Conversely, Zeng et al. recently indicated that NOTCH1 in fact promotes osteogenic calcification in human VIC [16]. Recent work, using induced pluripotent stem cell- (iPSC-) derived ECs in vitro, showed that* NOTCH1* haploinsufficiency disrupts the EC response to shear stress and unlocks proosteogenic and inflammatory network [10]. These disparate findings highlight the need for further studies in order to elucidate the pathological alterations due to NOTCH pathway attenuation. The NOTCH cascade is one of the possible regulators of OPG/RANKL/RANK system [15]. Osteoprotegerin (OPG) is a cytokine member of the TNF receptor superfamily and binds two ligands, one of which is RANKL (receptor activator of nuclear factor kB ligand), a critical cytokine for osteoclast differentiation [17, 18]. OPG-deficient (OPG−/−) mice develop severe osteoporosis and prominent vascular calcification at an early age [19]. OPG exhibits an inhibitor control on RANK and its ligand RANKL, which promotes skeletal demineralization and increases calcification of blood vessels. It is proposed that osteoprotegerin (OPG) being one of the key molecules in the ossification process may play an important role in aortic valve and vascular wall calcification [20, 21]. Changes in RANK, RANKL, and OPG gene expression have been observed in native stenotic and bioprosthetic valves [22]. However, inferences from these studies have been controversial, and the exact role of OPG/RANKL in aortic valve calcification remains to be established. Uncovering the genetic and environmental factors that regulate the OPG/RANKL/RANK system and the identification of patient subgroups with higher probability of aortic valve calcification constitute an important fundamental and clinical issue.

Accordingly, the aim of our study was to search for* NOTCH1* mutations in patients with AS and to assess the possible association between* NOTCH1* mutations and OPG/RANKL/RANK system in patients with different morphological variants of AS.

## 2. Materials and method

### 2.1. Ethics Statement

The study protocol was approved by the local ethics committee at the Federal North-West Medical Research Centre (Saint Petersburg, Russian Federation) before the initiation of the study, according to the principles of the Declaration of Helsinki. Written form informed consent was obtained from all participating patients.

### 2.2. Study Cohort

61 patients with severe aortic valve stenosis were selected from database of 530 patients with AS treated and observed in Almazov Federal North-West Medical Research Centre between 2010 and 2011, with a comparable distribution by TAV and BAV morphology and age.

The control DNA was obtained from 200 healthy donors without valvular heart diseases confirmed by transthoracic echocardiography (ECHO). Controls were randomly selected from relatively healthy bank employers [23]. However, 30% of the control group had hypertension and dyslipidemia were detected in of 33% of controls. The demographic and clinical characteristics of the groups are presented in Tables 1 and 2.

### 2.3. Echocardiography

All patients of study and control group underwent comprehensive 2-dimensional and Doppler transthoracic ECHO using the Vivid 7.0 system (GE, USA), according to the current guidelines [1, 24]. Criteria for severity of aortic valve stenosis included aortic valve area (AVA, cm^2^), calculated using the continuity equation; AVA indexed for body surface area (AVA/BSA, cm^2^/m^2^); and mean transvalvular pressure gradient and peak aortic jet velocity (*V*max). We included patients in our study if *V*max at the aortic valve was more than 4.0 m/s [1, 24]. Diagnosis of BAV was based on short-axis imaging of the aortic valve demonstrating the existence of only 2 commissures delimiting only 2 aortic valve cusps. Patients with known infective endocarditis and rheumatic disease as well as patients with left ventricular systolic dysfunction were excluded from the study.

### 2.4. Measurement of Circulating Biomarkers

In the previous pilot study we have demonstrated that OPG levels were increased in patients with AS while elevated sRANKL was found only in patients with BAV [25]. In the present study we measured the circulating biomarkers OPG and serum RANKL in 61 patients with severe AS and 32 sex- and age-matched individuals of the control group (Table 2). Peripheral venous blood was obtained at 8:00 AM after overnight fasting and refraining from smoking. Serum samples were immediately frozen and kept at −70°C until assay. The results were interpolated from the standard reference curve provided with each kit. Biomarkers of inflammation, lipid metabolism, and valvular calcification were examined. Serum OPG and serum RANKL levels were determined using a human Osteoprotegerin Instant ELISA kit (Bender MedSystems GmbH, Vienna, Austria) and the human sRANKL ELISA development kit (BIOMEDICA, Wien) according to the manufacturer's instructions. The results of the OPG assays were expressed as pmol/l. The OPG/sRANKL ratio was calculated for all patients. The lipid profile was performed in an autoanalyzer (Cobas Integra 400+), using commercially available kits (Roche Diagnostics). The results were interpolated from the standard reference curve provided with each kit. Intra-assay variation was 4% or less, interassay variation was 9%, and all laboratory work was undertaken by researchers who were blinded to the patients' clinical details.

### 2.5. Sequencing of the* NOTCH1 *Gene

DNA samples were collected from all patients with AS and from the control group. Genomic DNA was extracted from peripheral blood using a FlexiGene DNA purification kit (Qiagen, GmbH, Hilden, Germany). We applied a strategy of targeted mutation screening for 10 out of 34 exons of the* NOTCH1 *gene. Amplification of exons 10, 11, 12, 13, 20, 23, 24, 29, 30, and 34 was performed (primers available upon request). The choice of these specific exons was based on previously published reports on the implication of* NOTCH1* mutations in cardiac malformations, including BAV, aortic aneurysm, and left ventricular outflow track (LVOT) malformations [9, 26–28]. Mutation screening in patients and control groups was performed by direct sequencing of amplified fragments with an ABI capillary sequencer (Applied Biosystems, Foster City, CA, USA) using BigDye Terminator v3.1 mix (Applied Biosystems). The obtained sequences were analyzed and aligned using Geneious software; new and rare variants were checked against the control group and ExAC databases. Nucleotide numbering and mutation nomenclature were based on a reference* NOTCH1 *cDNA sequence (GeneBank Accession Number NG_007458.1, from NCBI). Functional prediction and annotation of nonsynonymous single-nucleotide variants in the human NOTCH1 gene were assessed based on the MetaSVM prediction obtained from the dbNSFP database [29]. MetaSVM is a support vector machine based prediction, which classifies amino acid substitutions as tolerated or damaging by incorporating deleteriousness scores produced by 9 individual algorithms: SIFT, PolyPhen-2, GERP++, Mutation Taster, Mutation Assessor, FATHMM, LRT, SiPhy, and PhyloP. Additionally, we present the CADD and PROVEAN results. The larger the CADD score is, the more likely the SNP has a damaging effect. Larger GERP++, PhyloP, and SiPhy scores correspond to more conserved sites: deleterious thresholds are written in brackets after the names of methods.

### 2.6. Statistical Methods

Statistical analysis was performed using Statistica for Windows ver. 10.0 (StatSoft Inc., Tulsa, OK, USA). Continuous data were tested for normality using the Kolmogorov-Smirnov test. Normally distributed data are expressed as mean (± standard deviation SD) and nonnormally distributed data as median (range). The significance of differences in mean values was assessed by one-way ANOVA with post hoc testing for multiple comparisons. Also, we used Kruskal-Wallis test between groups with nonnormally distributed data. A *P* value < 0.05 was considered to indicate statistical significance. Categorical variables are compared by Chi-square analysis or Fisher's exact tests and summarized by proportion in each category. Pearson's correlation test and simple regression analysis were used to analyze the association between data. Bonferroni correction was used to calculate a likelihood ratio test thresholds for genomewide (*P* < 0.05) and suggestive (i.e., one false positive per genome scan) significance. An age, systolic blood pressure adjusted multivariable regression analysis was performed to evaluate NOTCH1 nonsynonymous variants as predictors of AS and in separate analysis circulating levels of biomarkers were assessed as predictors to AS.

## 3. Results

### 3.1. Clinical Characteristics of Patients with Aortic Stenosis

In the main study group of AS (*n* = 61) there were no significant demographic and clinical differences between BAV (*n* = 31) and TAV (*n* = 30) subgroups (Table 2). The control group for DNA study was younger and had a significantly larger proportion of smokers compared with AS patients. Despite regular statin use by 59% of the patients with AS, the target total cholesterol level was not reached in most of patients. Approximately 90% of AS patients received concomitant therapy including beta-blockers (83%) and ACE inhibitors or angiotensin II receptor blockers for hypertension (36%). According to current guidelines, 46 symptomatic patients with AS underwent aortic valve replacement (75%); in each surgical patient, echocardiographic assessment of the aortic valve as bicuspid or tricuspid was confirmed intraoperatively. Echocardiographic parameters for the AS study cohort are presented in Table 3. All patients had AVA less than 1.0 cm, *V*max AV higher than 4.0 m/c, and a mean AV gradient more than 40 mmHg. There was no significant difference in echocardiographic parameters between patients in the BAV and TAV subgroups (Table 3).

### 3.2. Sequencing of the* NOTCH1 *Gene in Patients with Aortic Stenosis

**Figure 1:**
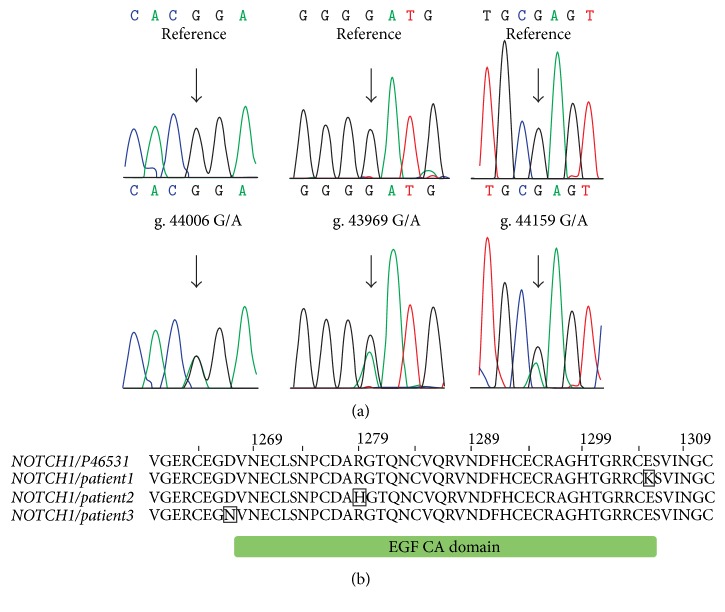
Localization of missense mutations in NOTCH1. (a) DNA sequences, characterized by location of the missense mutations in three patients in comparison with the reference sequence of NOTCH1 gene (NG_007458.1). (b) Multiple sequence alignment of wild-type NOTCH1 protein (Uniprot ID: P46531) and mutated variants located in patients. EGF CA domain is a calcium-binding epidermal growth factor-like domain of NOTCH1. The amino acid residues in boxes are mutated positions.

**Figure 2:**
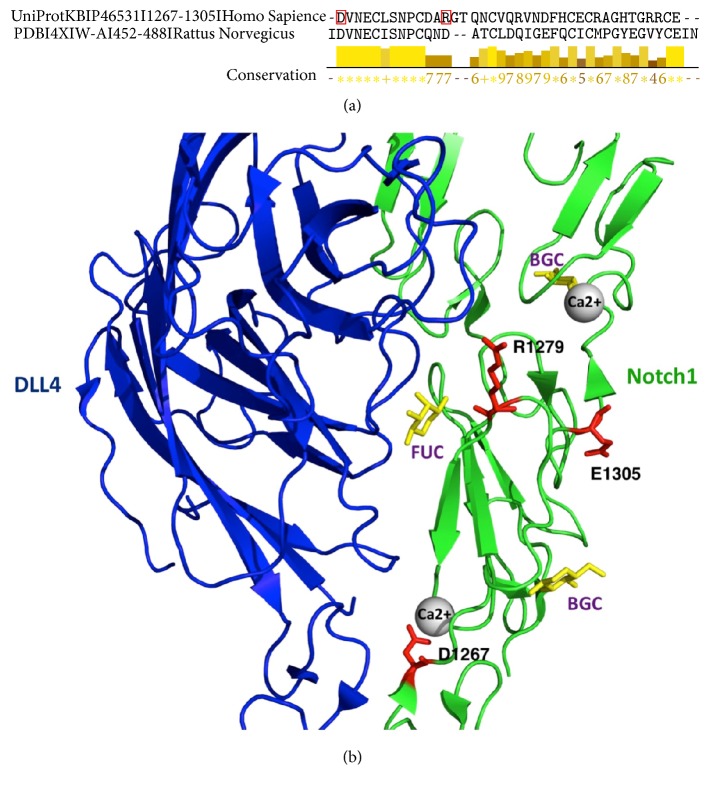
Structure of NOTCH1 EGF CA domain. (a) The sequence alignment of human NOTCH1 EGF CA domain with rat NOTCH1 EGF CA domain of known crystal structure (PDB ID: 4XLW Chain A) visualized in Jalview 2.8.2 [11]. Highly conserved residues (conservation score = 11) are marked as “*∗*” and identical residues (conservation score = 10) are marked as “+.” The amino acids in red boxes are mutated positions. (b) Three-dimensional structure of EGF modules of the NOTCH extracellular domain bound to DLL4 [12] visualized by PyMol. Structures of proteins are shown in cartoon representation. Calcium ions are represented as gray spheres. *β*-D-glucose (BGC) and *α*-L-fucose (FUC) molecules are highlighted in yellow. Residues of interest E1305, D1267, and R1279 are marked as sticks in red color.

Ten out of 34 exons of the NOTCH1 gene and adjacent intronic fragments were sequenced in the patients with AS and in the control group. We detected 6 genetic variants in the coding region of the studied exons, 3 of which lead to the amino acid change (Table 4). Two novel heterozygote mutations, E1305K located in exon 24 and D1267N located in exon 23, were found. Additionally, one nonsynonymous heterozygote variant R1279H previously reported by our group and others as a rare polymorphism was identified in both the study and control groups [28, 30]. However, this variant was considerably overrepresented in patients with AS compared with the control group without heart diseases (6 out of 61 and 4 out of 200, resp.; *P* < 0.01), an association that remained significant after adjustment for multiple testing. Since this variant may therefore represent a disease susceptibility allele, it was further analyzed together with two new missense variants. Missense SNVs in NOTCH1 gene were analyzed using dbNSFP metaserver. This method has shown that the replacement of glutamic acid (E) by lysine (K) in 1305 position of NOTCH1 protein is highly pathogenic and has a deleterious effect on protein function (Table 5). The substitution of aspartic acid by asparagine is predicted to be tolerated, but SIFT, PolyPhen2, LRT, Mutation Taster, PROVEAN, and CADD point to the deleteriousness of this mutation. The R1279H mutation is not damaging that was confirmed by all tools except for LRT and Mutation Taster. All investigated missense SNVs are located in highly conserved positions in the protein (their scores in GERP++ and SiPhy are higher than deleterious thresholds). Mutations D1267N, R1279H, and E1305K are located in Ca(2+)-binding epidermal growth factor (EGF) motif of the NOTCH extracellular domain bound to Delta-like 4 (DLL4) (Figure 1), according to the SMART diagram [31] of domains within* Homo sapiens* protein NOTCH1 (Uniprot ID: P46531). Two mutations D1267N and R1279H were found in NOTCH1/DLL4 interface and can be crucial in their protein-protein interaction (Figure 2). The mutation E1305K is positioned on the surface of protein and can be important in binding with other proteins. Moreover, 1267 and 1305 are the first and last positions in separate EGF domain and are highly conserved in other orthologous.

### 3.3. Serum Concentration of OPG and sRANKL

**Figure 3:**
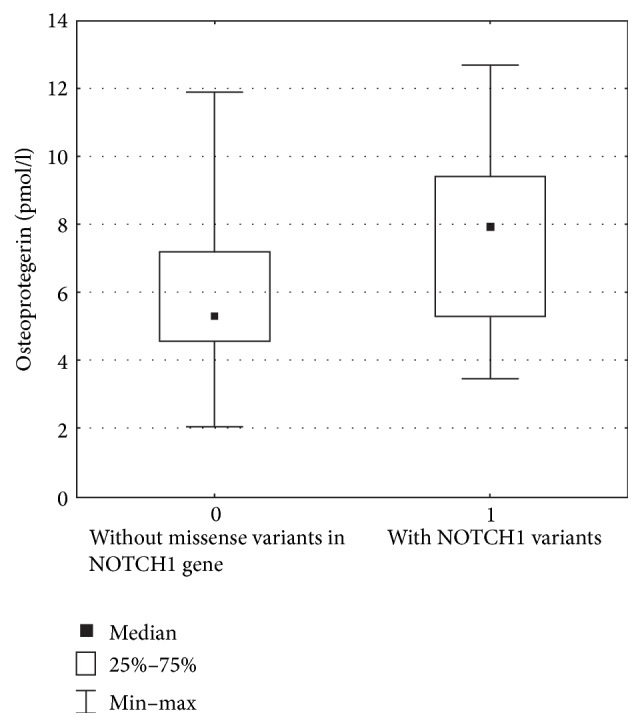
Box and whisker plots the distributions of serum OPG levels in patients with or without* NOTCH1* missense variants: 7.9 (5.3, 9.4) and 5.3 (4.6, 7.2), *P* = 0.044, comparable.

In AS patients, the OPG levels were significantly higher compared to those of the control group (Table 2). For sRANKL levels, a significant increase compared with controls was observed only in patients with BAV, not in those with TAV. Whereas there was no significant difference in the levels of either serum OPG or sRANKL between BAV and TAV subgroups of patients with AS, the OPG/sRANKL ratio was significantly higher in TAV compared with BAV (Table 2). In univariate analysis, sRANKL was positively associated with office systolic blood pressure level only in patients with TAV (*r* = 0.49; *P* < 0.01). Positive correlation was observed between age and the OPG/sRANKL ratio in all patients with AS (*r* = 0.30; *P* < 0.01). Of note, patients with* NOTCH1* missense variants (E1305K, D1267N, and R1279) had higher concentrations of OPG (Figure 3) and OPG/sRANKL ratio (*P* < 0.01). The results of the multivariable regression adjusted for age and systolic blood pressure revealed significant association of OPG and OPG/sRANKL ratio with* NOTCH1* missense variants (E1305K, D1267N, and R1279), respectively (*β*-coefficient = 0.290; *P* = 0.03 and *β*-coefficient = 0.240; *P* = 0.04). Mutations in* NOTCH1* gene were not associated with increased sRANKL concentration.

## 4. Discussion

We have shown here that* NOTCH1* rare variants are overrepresented in AS patients when compared with controls and also we have identified two new* NOTCH1* mutations. Furthermore, the functional prediction method dbNSFP identified the E1305K as pathogenic substitution, while D1267N and R1279H were considered tolerated. However, some in silico tools have predicted D1267N as deleterious variant. In addition, all missense variants described in* NOTCH1* gene have been found in Ca(2+)-binding EGF motif of the NOTCH extracellular domain bound to Delta-like 4. The Ca(2+)-binding sequence motif is coupled to a sequence motif that brings about beta-hydroxylation of a particular aspartate residue. Point mutations in EGF modules that involve amino acids which are Ca(2+) ligands result in the biosynthesis of biologically inactive proteins. The replacement of D by N in 1267 position may change the affinity of this residue to an ion Ca2+ and affects its binding to Jagged1 and DDL4 [32, 33]. The calcium binding by NOTCH EGFs is important for folding and ligand association [12]. The residue D^1267^ in human* NOTCH1* EGF domain corresponds to D^469^ in rat domain due to the sequence alignment (Figure 2(a)). D^469^ is a calcium-coordinating residue that is identical in all four NOTCH receptors [11]. On the other hand, this residue is crucial for Jagged1 recognition and participates in hydrogen-bond formation with DLL4. Second, this association was independent of the valve morphology (BAV versus TAV). Finally, we show that carriers of* NOTCH1* variants had higher levels of OPG and a higher OPG/RANKL ratio compared to carriers of the common allele. Taken together, these findings suggest that genetic variations in the NOTCH pathway may affect aortic valve calcification through the OPG/RANKL pathway.

The associations of* NOTCH1* mutations with various cardiovascular phenotypes, such as BAV, aortic aneurysm, and aortic coarctation, have been reported earlier [9, 26–30, 34]. In the present study, we extend the spectrum of* NOTCH1*-associated cardiac disorders to calcified AS, independently of the BAV or TAV morphology. Beyond genetic factors, we show that age and arterial hypertension were independent predictors for OPG/RANKL/RANK system activation in patients with TAV. This notion is in line with OPG playing a key role in the acquisition of an osteogenic phenotype in both vascular and valvular cells.

In our study we found higher serum OPG levels in BAV and TAV patients with calcific aortic stenosis than in controls, which corresponds with previously published data [35–37]. This is however not in agreement with the report of Adamczyk et al., where no significant differences in OPG concentration were found between patients with and without degenerative AS [38]. One possible explanation for this difference could be the high prevalence of coronary artery disease and thus active atherosclerotic processes that might have been associated with higher OPG levels in their control group. In our study cohort, only 11 out of 46 AS patients with catheterization with angiography prior to valve replacement had concomitant CAD, and there were no patients with documented CAD in the control group. The lack of difference in OPG levels between BAV and TAV is also in contrast to the study by Nagy et al., which measured OPG in plasma instead of serum [39]. Taken together, these demographic and methodological differences in populations might explain some of the controversy surrounding divergent reports on OPG levels and calcification process.

The serum sRANKL concentration in our study was significantly increased only in the AS subgroup with BAV. This difference might attribute to the different genetic and molecular mechanisms of TAV and BAV calcification since we did not observe any difference in clinical and echocardiographic parameters between these two subgroups. The protective role of increased sRANKL is widely debated with several reports illustrating the association of increased sRANKL level with beneficial cardiovascular prognosis, while some opposing results also have been published [40, 41]. For example, the levels of circulating RANKL in relation to OPG levels (RANKL/OPG ratio) were significantly correlated with a decreased AVA in a study of 46 patients with AS [39]. Taken together, the observed increased serum sRANKL levels and OPG∖sRANKL ratio in BAV patients in our study warrant further investigation.

Our study had several limitations. First, we were detecting OPG and RANKL concentration in serum knowing that these circulating levels reflect their production in many tissues and organs beyond the aortic valve, which makes it difficult to specify the source of elevated OPG/RANKL ration. A second limitation was the inability to establish familial segregation or de novo origin of the* NOTCH1* mutations and a limited sequencing of the genes, thus, making the disease causality of identified variants not strictly proven. Another limitation is the group size of 61 pts with severe AS. Further studies on a larger group of patients with different degrees of severity of AS are required.

## 5. Conclusion

Our study showed that mutation in* NOTCH1* might play an important role in the formation and progression of aortic valve calcification, associated with a dysregulation of OPG∖RANKL∖RANK system, due to modulation of the ligand-binding site in NOTCH1 by calcium affinity. These results add to the current understanding of the pathogenesis of aortic calcification and may represent clinical utility, especially in terms of prognosis of patents with aortic stenosis.

## Figures and Tables

**Table 1:** Clinical characteristics of patients in aortic stenosis and control groups.

	Patients with AS M ± SD	Patients with BAV M ± SD	Patients with TAV M ± SD	DNA control group	*P* value^*∗*^ versus DNA control group	*P* value TAV versus BAV patients
	(*n* = 61)	(*n* = 31)	(*n* = 30)	(*n* = 200)		
Age, years	57.1 ± 6.4	55.6 ± 8.0	58.8 ± 3.7	46.7 ± 8.7	<0.01	0.9
Gender, m : f	1.3 : 1	1.8 : 1	1 : 1	1 : 1.1	0.5	0.25
BMI, kg/m^2^	28.9 ± 4.9	28.1 ± 3.8	30.0 ± 5.9	29.1 ± 4.5	0.48	0.10
Arterial hypertension, *n* (%)	50 (82%)	22 (71%)	28 (93%)	60 (30%)	<0.01	0.05
Systolic BP, mmHg	170 ± 29	163 ± 31	176 ± 24	140 ± 16	<0.01	0.11
Diastolic BP, mmHg	98 ± 15	95 ± 16	101 ± 13	90 ± 11	<0.01	0.12
Diabetes mellitus, *n* (%)	10 (16)	5 (9.7)	5 (13.3)	3 (1.5)	<0.01	0.97
COPD, *n* (%)	12 (19.7)	5 (16.1)	5 (16.7)	10 (10)	0.67	0.86
Smoking, *n* (%)	14	7 (22.6)	7 (23.3)	102 (51)	<0.01	0.97
Total cholesterol, mmol/l	5.6 ± 1.37	5.84 ± 1.27	5.31 ± 1.45	5.8 ± 1.51	0.3	0.20
HDL-C, mmol/l	1.46 ± 0.41	1.46 ± 0.31	1.47 ± 0.54	1.40 ± 0.41	0.46	0.97
LDL-C, mmol/l	3.62 ± 1.49	3.73 ± 1.36	3.50 ± 1.50	3.64 ± 1.1	0.99	0.63
Triglycerides, mmol/l	1.62 ± 0.87	1.65 ± 0.82	1.59 ± 0.97	1.67 ± 1.43	0.82	0.85
Medication, *n* (%)	54 (88)	27 (87)	27 (90)	47 (24)	<0.01	0.98
ACE inhibitors /ARB, *n* (%)	22 (36)	13 (41.9)	9 (30)	47 (24)	0.02	0.61
Beta-blockers, *n* (%)	51 (84)	26 (83.9)	25 (83.3)	0	—	0.92
CCB, *n* %	3 (5)	2 (6.5)	1 (3.3)	0	—	0.85
Statins, *n* (%)	36 (59)	18 (58.1)	18 (60)	0	—	0.98

DNA control group: healthy donors, including for control DNA; BMI: body mass index; BP: blood pressure; COPD: chronic obstructive pulmonary disease; HDL-C: high-density lipoprotein cholesterol; LDL-C: low-density lipoprotein cholesterol; ARB: angiotensin II receptor blockers; ACE: angiotensin-converting enzyme inhibitors; CCB: calcium-channel blocker. ^*∗*^Patients with AS.

**Table 2:** Clinical characteristics of patients with aortic stenosis and control group for analysis of the circulating biomarkers.

	Patients with AS M ± SD	Patients with BAV M ± SD	Patients with TAV M ± SD	Control group for circulating biomarkers	*P* value^*∗*^ versus control group
	(*n* = 61)	(*n* = 31)	(*n* = 30)	(*n* = 32)	
Age, years	57.1 ± 6.4	55.6 ± 8.0	58.8 ± 3.7	57.5 ± 4.6	0.81
Gender, m : f	1.3 : 1	1.8 : 1	1 : 1	1 : 1	0.68
BMI, kg/m^2^	28.9 ± 4.9	28.1 ± 3.8	30.0 ± 5.9	26.2 ± 3.9	<0.01
Arterial hypertension, *n* (%)	50 (82)	22 (71)	28 (93)	10 (31)	<0.01
Systolic BP, mmHg	170 ± 29	163 ± 31	176 ± 24	133 ± 16.5	0.26
Diastolic BP, mmHg	98 ± 15	95 ± 16	101 ± 13	86 ± 9.0	0.14
Total cholesterol, mmol/l	5.6 ± 1.37	5.84 ± 1.27	5.31 ± 1.45	5.22 ± 0.84	0.84
HDL-C, mmol/l	1.46 ± 0.41	1.46 ± 0.31	1.47 ± 0.54	1.49 ± 0.33	0.33
LDL-C, mmol/l	3.62 ± 1.49	3.73 ± 1.36	3.50 ± 1.50	3.25 ± 0.89	0.89
Triglycerides, mmol/l	1.62 ± 0.87	1.65 ± 0.82	1.59 ± 0.97	1.21 ± 0.85	0.54
OPG (pmol/l) M ± SD	6.3 ± 2.4	6.2 ± 2.3^*∗∗*^	6.4 ± 2.6^*∗∗∗*^	4.8 ± 1.8	<0.01
sRANKL (pmol/l) M ± SD	0.45 ± 0.17	0.48 ± 0.18^*∗∗∗*^	0.42 ± 0.17	0.38 ± 0.12	0.06
ОPG/sRANKL	16.7 ± 11.3	13.9 ± 5.6	19.7 ± 14.8	14.4 ± 9.6	0.3

HDL-C: high-density lipoprotein cholesterol; LDL-C: low-density lipoprotein cholesterol; BMI: body mass index; BP: blood pressure. ^*∗*^Patients with AS; ^*∗∗*^*P* = 0.02 value versus control group; ^*∗∗∗*^*P* < 0.01 value versus control group.

**Table 3:** Echocardiographic parameters from patients with aortic stenosis.

	Patients with BAV M ± SD	Patients with TAV M ± SD	*P* value
AVA, cm^2^	0.84 ± 0.22	0.86 ± 0.18	0.73
AVA/BSA, cm^2^/m^2^	0.43 ± 0.1	0.45 ± 0.1	0.78
Peak aortic velocity, m/s	4.69 ± 0.68	4.5 ± 0.59	0.28
Mean pressure gradient, mmHg	53.8 ± 16.1	51.4 ± 16.1	0.61
EF_Simpson_, %	62.8 ± 6.7	62.5 ± 6.2	0.90
LVEDD, mm	49.4 ± 6.5	50.4 ± 6.5	0.56
Ascending aorta, mm	38.2 ± 5.2	38.5 ± 7.6	0.87
Aortic sinus, mm	36.9 ± 4.8	35.3 ± 3.8	0.19
LVMM, g	328.1 ± 127.5	307.1 ± 79.2	0.52
LVMI, g/m^2^	165.1 ± 51.8	157.8 ± 38.1	0.61
RWT, mm	0.55 ± 0.1	0.55 ± 0.2	0.74

AVA: aortic valve area; AVA/BSA: aortic valve area indexed for body surface area; aorta *V*max: antegrade velocity across the narrowed aortic valve; P-mean: the mean transvalvular pressure; EF: ejection fraction; LVEDD: left ventricle end diastolic diameter; RWT: relative wall thickness; LVMM: left ventricular myocardial mass; LVMI: indexed left ventricular mass.

**Table 4:** Genetic variants in NOTCH1 gene in patients with AS and control population.

Exon	Gene position NG_007458.1	Protein position	New/reported	Patients	Control group	MAF ExAC
Missense variants in NOTCH1 gene						
24	44159 G/A	E1305K	New	5/61	0/200	—
23	43969 G/A	D1267N	New	2/61	0/200	—
23	44006 G/A	R1279H	rs61751543	6/61^*∗*^	4/200	0.02

Synonymous variants in NOTCH1 gene						
34	53339 G/A	P2097P	rs201987555	1/61	0/200	0.01
34	53602 С/T	D2185D	rs2229974	31/61^*∗∗*^	60/200	0.61
34	53696 G/A	P2216P	rs3812596	4/61	0/200	0.01

^*∗*^ *P* < 0.01, ^*∗∗*^*P* < 0.01.

**Table 5:** Functional prediction of missense variants in NOTCH1 by sequence-based computational methods.

Mutation	E1305K	R1279H	D1267N
SIFT	D	T	D
Polyphen-2	T	T	D
LRT	D	D	D
Mutation Taster	D	D	D
Mutation Assessor	M	N	L
FATHMM	D	T	T
PROVEAN	D	T	D
CADD (>15)	29.0	23.5	28.5
GERP++ (>4.4)	4.64	4.03	5.23
PhyloP (>1.6)	0.84	0.85	0.85
SiPhy (>12.17)	16.49	13.63	17.78
dbNSFP	D	T	T

For SIFT, Polyphen-2, LRT, Mutation Taster, Mutation Assessor, FATHMM, PROVEAN, and dbNSFP (MetaSVM), we used the following abbreviations: D: damaging; T: tolerated; H: high; M: medium; L: low; N: neutral.
